# Development and use of a research productivity assessment tool for clinicians in low-resource settings in the Pacific Islands: a Delphi study

**DOI:** 10.1186/s12961-016-0077-4

**Published:** 2016-01-29

**Authors:** Alec J. Ekeroma, Boaz Shulruf, Lesley McCowan, Andrew G. Hill, Tim Kenealy

**Affiliations:** South Auckland Clinical Campus, University of Auckland, Middlemore Hospital, Auckland, New Zealand; Office of Medical Education, Faculty of Medicine, University of New South Wales, Sydney, New South Wales Australia

**Keywords:** Clinician researchers, Pacific Islands, Research indicators, Research performance

## Abstract

**Background:**

Research performance assessments have proliferated, but research indicators for use amongst clinicians in poorly resourced countries have been ill-defined. The aims of the present paper were to determine a set of indicators as determined by clinician participants from the Pacific Islands and a panel of research experts for use in the performance assessment of clinicians.

**Methods:**

Two focus group discussions, one for nurses and one for doctors, were used to obtain the views of 28 Pacific Island clinicians of the BRRACAP Study about what the research indicators should be. A modified Delphi survey was used to obtain a consensus amongst 19 research experts, with Pacific Island research experience, as to what the indicators should be and then to rank these in terms of importance. A survey of the participants obtained data on the research tasks/actions performed 20 months after the initial research workshop. A resultant tool comprising of 21 indicators was used to assess the performance of 18 Pacific participants.

**Results:**

The Pacific Island clinicians determined that research was important and that performance should be measured. They identified research indicators that could be used in their settings and ranked their importance using a points system. The panel of experts identified implementation of research findings, collaborations and actual change in practice as more important, with bibliometric measurements low down in the scale. Although only 64 % of the 28 BRRACAP Study participants returned the questionnaire, 39 % of those performed more than half of the 21 indicators used. Of the 18 Pacific clinicians assessed, 7 (39 %) performed 10 or more tasks.

**Conclusions:**

A research performance assessment tool was developed using process and output indicators identified by Pacific clinicians and a panel of research experts. The tool, which placed emphasis on process and outputs that were not bibliometric based, proved useful in assessing the performance of Pacific clinicians working in a low-resource setting.

**Electronic supplementary material:**

The online version of this article (doi:10.1186/s12961-016-0077-4) contains supplementary material, which is available to authorized users.

## Background

Measuring institutional and individual research performance is required for resource allocation decisions, professional progression and, most importantly, an investment in quality [1]. In addition, measuring research productivity increases the level of research, which is key to improving standards of living and poverty reduction [2]. Different countries have developed their own performance measures such as that of the Research Excellence Framework in the United Kingdom [3], the Excellence in Research for Australia [4], and the Performance-Based Research Fund in New Zealand [5]. The main difference between the REF, ERA and the PBRF is that the PBRF attempts to measure the individual performance of academic staff by including other markers of research activity such as peer esteem and contributions to the research environment [6].

Since the evolution of the citation index in the 1950s, bibliometrics have increasingly been used by scientists and policymakers to assess research productivity [7]. Whereas there is an agreement to measure individual research performance, there is no consensus as to which tools are valid and consistent [8, 9]. Further, there is a dearth of suggestions as to which tools to use to measure research productivity of part-time staff [10], such as clinicians, who are expected to perform clinical research mostly in their own time [11], especially in low- and middle-income countries (LMICs) where there is a growing drive by funders to measure effectiveness of capacity-building initiatives [12, 13] and by academic institutions to measure individual research productivity [14]. Although there is an abundance of studies using bibliometric indices to assess the health research performance of countries [15] or disciplines [16] within health systems in LMICs, there is only one paper which attempted to measure individual research productivity in these settings [13]; however, these measures were for those with a doctorate degree from international universities.

The general lack of well-functioning health systems, research skills, access to the literature, research funding, protected time for research [13, 14, 17–19], and the importance of cultural context would mean that the yardstick used in well-developed countries cannot be applied in LMICs. Wooton et al. [10] developed a “*generalizable method for measuring individual research productivity*”, but the measures were confined to research output and, of the 12 papers reviewed, only one was from an LMIC. A performance assessment tool should ideally include both process and output factors such as those in the Performance-Based Research Fund [5]. In addition, the input of those to be measured in the identification of relevant context markers of research productivity and in the design of the assessment tool is needed for buy-in and sustainability.

The aims of the present paper were to develop a research performance-appropriate tool for clinicians working in low-resource settings such as those in the Pacific Islands. The tool is then to be used to assess the research productivity of 28 clinicians of the BRRACAP Study [20].

## Methods

### The BRRACAP Study – Pacific clinician participants

The 28 study participants were clinicians selected from the leadership of the reproductive health services in six Pacific countries and criteria of selection have been previously published [17, 20]. After a research workshop in March 2013, they were supported with assigned research mentors to work on research and clinical audit projects. A further 1-day research workshop was provided 4 months later, which was attended by 12 of the participants. Online support through ResearchGate, LinkedIn and Facebook supplemented email support from mentors. The participants agreed to a measurement of their research productivity 2 years after the initial workshop. Ethics approval was obtained from the University of Auckland Human Participants Ethics Committee (Ref. No. 8373).

### Developing a research productivity measure for clinicians in low-resource settings

#### By the clinical participants

Focus group discussions (FGDs) were used to develop appropriate indicators of research productivity for clinicians in the Pacific. Emphasis was placed on what was achievable in a low-resource setting. Two FGDs were held for an hour in March 2013 during a 1-week research workshop; one FGD was for nurses and midwives and the second FGD was for doctors, with five and seven participants each, respectively. The FGD was facilitated by AE and was audio-recorded and transcribed. The aims of the FGD were to determine the research outputs/markers that were appropriate for their low-resource settings. Four questions were discussed:

1. Is research/clinical audit important in your work?
2. Is it important to measure research performance of clinicians and why?
3. How should we measure research performance?
4. Who should do the measuring?

Findings from the two FGD were then shared with all the participants the next day, where a further discussion was held to refine the weight of importance given to the identified markers of research productivity. All the participants agreed to have their research activities measured.

#### By a panel of experts

A modified Delphi technique [21] was used to develop two survey tools to establish a consensus among identified research experts for the most appropriate research indicators for the Pacific Islands. The experts, who all had research experience in the Pacific Islands, were identified by a search on Google Scholar and from the bibliography of a paper published on research in the Pacific Islands [15]. The two main criteria were that they had a track record of publications and that they had either performed or led research teams in the Pacific Islands. A total of 33 experts were invited, and 19 accepted participation; nine of the panel members were Professors, and three were Associate Professors, whereas 10 were based in Australia, seven in New Zealand, one in Papua New Guinea, and one in the United States.

In the first questionnaire (Additional file 1), the panel was shown research performance indicators and their weighting as determined by the clinicians of the BRRACAP Study from the FGDs. They were asked to rank those as relevant for a low-resource setting in the Pacific Islands and to provide comments. They were also asked to suggest any other indicators of research performance as they saw fit for a low-resource setting. These were collated into 21 indicators for individual performance, and those suggestive of institutional or organisational indicators were excluded. The ranking of all items were entered into an Excel spreadsheet and the weighting developed as a scale of 0 (not relevant), 1 (somewhat irrelevant), 2 (somewhat relevant), and 4 (very relevant). It was an arbitrary score, but it was simple and appropriate for this study.

In the second and final Delphi questionnaire (Additional file 2), the panel was shown how they ranked the indicators identified by the BRRACAP Study participants. The panel was also shown a list of 21 ‘other research performance indicators’ that had been suggested from the first questionnaire and should be considered for the low-resource setting. Individual panellist’s justification for each of the 21 indicators was also disclosed. They were asked to rank the extra list in order of importance and to provide further comments.

### Survey of participants

The Delphi panel of experts were unanimous in using a hybrid of research indicators from both the BRRACAP Study participant’s responses and the panel’s assessment in developing a tool to assess the research performance of clinicians working in low-resource countries.

A survey of the 28 BRRACAP Study participants was then performed to ascertain their research productivity from March 2013 to December 2014 (Additional file 3). The survey tool was developed using a pragmatic set of 21 research indicators that includes those identified by the Delphi panel and the study participants themselves. The replies from the participants were interpreted and collated against the 21 research indicators on an Excel spreadsheet. The replies about the research activities were not verified, i.e. an audit project had been started, but evidence was not required. For simplicity, the participant either delivered or did not deliver on a research indicator/activity and each indicator was awarded one point. Ranking of participants’ performance using the sum of indicator points performed was compared to the ranking value as determined by the panellists, using Spearman’s rank correlation test for a comparison of the two non-parametric variables.

## Results

### Focus group discussion with the BRRACAP study participants

#### Emerging themes

The FGDs resulted in better understanding of barriers in conducting research in the Pacific Islands and how the participants felt about research and clinical audit in general. Each focus group identified the most important research issues as it pertains to their professional roles and responsibilities. There was a lot of common ground between the two groups whereas the differences were mainly in the weighting given to different research indicators.

#### Research is important to clinicians and clinical practice

Both groups agreed that clinical research and audit were important to clinical practice. That research was needed to see whether a change in practice was needed. There was some hesitancy as to how much research should be performed by clinicians who are also busy with clinical work. There was also the issue of lack of research skills by clinicians and the lack of support that is required by them to do research.

#### It is important to measure research output

Both doctors and midwives/nurses groups thought it was important to measure research performance/output. The academics thought it important for job security.

#### There are many research indicators

There were many research indicators identified and the two groups preferred them in a list included in their job description or standards of practice. Research indicators ranged from publications to writing annual reports for the Ministry of Health.

The groups agreed that the easiest way to measure research performance was to determine the number of ‘research points’ for each agreed research indicator or activity. There was an argument that research points should be awarded to clinicians who do the clinical work while giving others the time to do research. There was also a view that whatever research points or value the New Zealand clinicians receive for a particular research activity should be doubled for clinicians in the Islands as it was more difficult to do research there compared to New Zealand, where there was easier access to research support and resources. There was also the view that research points should be awarded for writing funding proposals even if the application was not successful.

#### Established organisations should do the measuring

The nurses and midwives group thought that, since there is a nursing council in each of the Island countries mandated to regulate the nursing and midwifery profession and practice, it should be these organisations that should set policies on nursing research practice. Further, these organisations should also perform the measuring of nursing research activity. Those with academic appointments have research performance requirements dictated by the academic institution and thought that the measuring should be preceded by a policy on clinical research followed by its embedding in standards of practice.

On the other hand, it was felt that, in the absence of research policies and measuring by the nursing councils and nursing/midwifery organisations (absent in most small countries), regional professional organisations, such as the Pacific Society for Reproductive Health, should do the measuring.

#### Indicators and points for each activity

In identifying the research indicators, the participants asked or referred to what was used as research indicators in academic institutions in New Zealand. For example, there were questions whether a general meeting or ward round could be considered a research activity, referring to points they would collect for continuous professional development activities. Therefore, it was easier to rank the importance of each activity by using points that were used in continuous professional development programmes. Table 1 summarises the research indicators and their respective importance in points as identified by the participants of the two FGDs and later refined by all the participants.

**Table 1:** Research performance indicators and weighting identified by Pacific clinicians at focus group discussions

Midwives/Nurses points	Medical doctors points	Research performance indicators
20		Regional recognition as a researcher
10	10	Research publication in a peer-reviewed journal
10		Lead author of a research-based practice guideline that is endorsed or approved
10	10	Successful at obtaining research funding
10	10	Submit a research proposal
5	10	Presentation at a regional research conference
5	5	Completion of a clinical audit project
5	1 point/hr	Attending research conference
3	5	Annual report writing and recommendations
2	10	Contribute/revise local practice guidelines
1 point/hour	1 point/hour	Participation in journal clubs
1 point/hour	1 point/hour	Organizing research meetings
1 point/hour	1 point /hour	Teaching or mentoring research students
1 point/hour	1 point /hour	Interacting with mentor

### Panel of experts

#### Delphi survey research indicator rankings

The expert panel ranked the importance of research indicators commonly used in high-resource countries in their importance to themselves as academics and to clinicians working in low-resource settings such as the Pacific Islands (Table 2). Members of the panel suggested additional indicators or surrogate markers of research productivity to use in the assessment of research performance. These are summarised in Table 3.

**Table 2:** The panel ranking of research indicators commonly used in developed settings and how they should apply to clinicians in low-resource settings (n = 19)

Research performance indicators used to assess performance of academics in high-resource countries	VR ++++	SR ++	SI +	NR 0	Scale	Rank
Research collaborations	15	2			3.76	1
National recognition	14	3			3.65	2
Research supervision	15	2	3		3.35	3=
Contribution to the research environment, e.g. research meetings	13	1	3		3.35	3=
Conference presentations	7	10			2.82	5
Research funding received	7	7	2	1	2.59	6
International recognition	6	8	3		2.53	7
Number of publications	6	6	3	2	2.29	8
Reviewer	3	7	7		1.94	9
Peer esteem, e.g. journal editor	3	7	6	1	1.88	10
Article citations	3	5	8	1	1.76	11
Creative works	2	7	3	5	1.47	12
Books published		5	10	2	1.18	13

VR, Very relevant; SR, Somewhat relevant; SI, Somewhat irrelevant; NR, Not relevant.

Not all 19 panellists answered every question.

**Table 3:** Additional research indicators for clinicians in low-resource countries as identified and ranked by the expert panel (n = 19)

Additional research performance indicators	VR	SR	SI	NR	Score	Rank
Implementation/translation of research findings	16				4.00	1
Impact or change as a result of research	14	2			3.75	2
Policy briefs and media interaction	13	2			3.73	3
Position as principal investigator in research design and priorities	13	3			3.63	4
Relevance of research	12	4			3.50	5
Collaborations	11	5			3.38	6
Quality assurance projects	10	5			3.33	7
Community engagement and recognition	11	5	1		3.24	8
Advocacy	8	4	1		3.15	9
Development of Pacific or ethnic standards	7	6	2		2.80	10

VR, Very relevant; SR, Somewhat relevant; SI, Somewhat irrelevant; NR, Not relevant.

Not all 19 panellists answered every question.

### Themes from Delphi survey

#### Impact of research or implementation of findings is important

There was a consensus that clinicians in the low-resource settings in the Pacific Islands should perform research. However, the research needs to be ‘useful’ research – research that will have an impact on clinical practice or that the findings of which are implementable in the local setting.

“*Effecting improvement in practices and policies is a priority. Research to inform improved health outcomes in local settings (I think this is important in all settings, but especially so where there is a high burden of both infectious diseases and NCDs)*”*.*

“*Leadership in pushing research evidence into the policy arena. This is a real sticking point for all researchers – we are good at collecting data, but leave it there and it is not good sitting in journals in contexts where that evidence may play a role in promoting safer, more effective, equitable etc., practice or environments*”.

“*Local evidence generated relevant to local settings is especially important for informing locally-relevant health policy. This reduces the risk of imported responses that ‘miss the mark’ in terms of local cultural, social and spiritual understandings of health*”.

“*The extent to which the research contributed to clinical practice, programs and policies the setting in which the person works and in the Pacific*”.

#### Collaboration (inter-disciplinary and regional collaborations)

Research collaborations have consistently featured high in the panellists ranking both as an indicator in their setting, but also for those working in low-resource settings. This was again highlighted as an additional indicator. There was a feeling that researchers in the Pacific Islands do not have the necessary skills or resources and need support from better-resourced researchers. Collaborations would not only offer support but may also bring funding.

“*In context where resources are limited, the collaboration between disciplines (e.g. education and health) can make an important contribution to the overall health of the community*”.

“*Participation with national and regional colleagues in formulating important research questions, conducting studies and transforming practice, programs and policies*”.

“*Indicator of activity involving a wider group with mix of expertise is required to address major LMIC MDG-relevant issues*”.

“*Clinicians are still highly respected in society (Pacific and non-Pacific) and their voice is not heard enough. Free media, working with other researchers (being realistic about time and capacity constraints) and developing a track record in a field is vital as part of the wider efforts for change and development in the clinical and public health fields*”.

#### More Pacific clinicians as principal investigators

There was a strong feeling that, although collaborations were important, Pacific clinical researchers should lead research initiatives and plan studies.

“*Investment in research methods or translation and interpretation of research to benefit the field and the clinicians career development*”.

“*Pacific clinicians involvement in research planning, priority setting and research design – both at a project level and at a research governance level (e.g. involvement in ethics committee, research councils etc.)*”.

“*Need to identify contributions to study design, measures, processes and role in co-authored publications. As opposed to merely facilitating or ‘opening doors’ for outsiders*”.

“*Pacific clinicians to take greater role in the preparation of journal or conference and other forms of presentation – this may require additional support and training as writing and presentation are not traditionally part of the clinical role*”.

#### Measure of social media and other interactions

There was a feeling that, as long as research findings were communicated effectively, such as in social media, policy briefs, etc., then the fact that a journal publication was not achieved was of little importance.

*“Other forms of engagement and publication, which are directed at a more popular and general audience….measure of social media and other interactions with published materials*”.

“*Beyond formal publication… do researchers produce other types of outputs that are more accessible to a broader community?*”

### The research performance tool

The panel of experts and the BRRACAP Study participants identified indicators of research activity for use in the low-resource settings in the Pacific Islands. The indicators were a mixture of what can be considered, processes (e.g. research meetings, journal clubs, collaboration), outputs (e.g. publications, completed guidelines, dissemination), and outcomes (e.g. changed/improved practice). The panellists were in consensus that a hybrid measure of indicators identified by themselves and the participants should be used in a tool to assess research performance. Tables 2 and 3 were combined into Table 4 by keeping only the 16 top ranked research indicators.

**Table 4:** All research indicators for resource-poor countries (Tables 2 and 3 combined)

Research performance indicators for clinicians in resource-poor countries	VR ++++	SR ++	SI +	NR 0	Scale	Rank
Implementation/translation of research findings	16				4.00	1
Research collaborations	15	2			3.76	2
Impact or change as a result of research	14	2			3.75	3
Policy briefs and media interaction	13	2			3.73	4
National recognition	14	3			3.65	5
Position as principal investigator in research design and priorities	13	3			3.63	6
Relevance of research	12	4			3.50	7
Research supervision	15	2	3		3.35	8=
Contribution to the research environment, e.g. research meetings	13	1	3		3.35	8=
Quality assurance projects	10	5			3.33	10
Community engagement and recognition	11	5	1		3.24	11
Advocacy	8	4	1		3.15	12
Conference presentations	7	10			2.82	13
Research funding received	7	7	2	1	2.59	14
International recognition	6	8	3		2.53	15
Number of publications	6	6	3	2	2.29	16

VR, Very relevant; SR, Somewhat relevant; SI, Somewhat irrelevant; NR, Not relevant.

Not all 19 panellists answered every question.

### Survey of BRRACAP study participants

Of the 28 participants of the BRRACAP study surveyed, 18 (64 %) completed the questionnaire after three reminders by email. Of the 18 participants, 16 interacted with their research mentor and 15 had started an audit project (Table 5).

**Table 5:** Number (%) of BRRACAP Study participants who achieved a specific research indicator from 21 indicators – for respondents and for the whole group

Number of research activities/indicators	Research indicator/activity	Number (%) of participant respondents that achieved indicator	Percentage of all participants inclusive of non-respondents
		(n = 18)	(n = 28)
1	Interacting with research mentor	16 (89)	57
2	Started an audit project	15 (83)	54
3	Changes in clinical practice as a result of being engaged in the study	13 (72)	46
4	Guideline revision	12 (67)	43
5	Teach research	8 (44)	29
6	Advocated for change as a result of research	8	
7	Interaction with others about research or as a result of research	8	
8	Attend conference	7	
9	Recognised by peers or management as a researcher	5	
10	Submitted an ethics application	5	
11	Started or participated in a Journal club	5	
12	Presented a research paper	5	
13	Completed an audit project	5	
14	Called a research meeting	4	
15	Supervised research	4	
16	Received an award	3	
17	Community engagement on research	3	
18	Received research funding	2	
19	Collaborations	2	
20	Publication in peer-reviewed journal	2	
21	Manuscript review	1	

The top 11 research performers were identified by the sum of the number of indicators from they completed. When the value of each indicator as ranked by the expert panel was used, two participants moved from numbers 13 and 15 to numbers 10 and 11 (Table 6).

**Table 6:** Performance of the top 11 participants of the BRRACAP Study of the 18 who completed questionnaires

Top 11 performers	1	2	3	4	5	6	7	8	9	10	11
Sum of activities (n = 21)	18	18	12	12	11	10	10	9	7	7	7
Score value	41	40	27	26	30	22	25	19	16	19	16

The Spearman’s rank correlation coefficient between the two ranking methods was 0.95454 and the two-tailed value of *P* is 0, which is >99 % significant.

## Discussion

This is the first study looking at a formal measure of research performance by clinicians in low-resource countries. The clinicians participating in the BRRACAP Study understood the importance of clinical research and audit in improving practice and equally, the importance of measuring research performance and output. They were not certain about what research activities or indicators were and how each would apply to their setting, although they had some idea of how much each one should be worth in ranking or importance. They were certain that professional organisations should perform the measuring of research activity within a determined policy framework. The Delphi survey of experts ranked research collaborations, national recognition, supervision and contributions to the research environment as important indicators in low-resource countries, whereas the number of publications, which was an important output in high-resource countries, was ranked lower. The panel also identified additional indicators, which emphasised the importance of conducting relevant research, and which are translated and implemented to cause an impact in practice and policy. The survey of the 28 BRRACAP Study participants with a response rate of 18 (64 %) showed that 16 had interacted with a research mentor and 15 had started an audit project, although only five had completed one in the 21 months since the first research workshop. Five had presented a research/audit paper and two had manuscripts accepted for publication in a peer-reviewed journal. The top five clinicians achieved more than half of the 21 research indicators and the top 11 achieved seven or more.

It is important to construct research assessment models for clinicians in low-resource settings that are inclusive of the collective view of research experts and local clinicians, acknowledging the importance of context in which the research is performed [14, 22, 23] and that the dimensions of research performance is in keeping with the functions and roles expected of the individuals or groups assessed [11]. Whereas bibliometric indicators have been the foundation of research evaluations in high-resource countries for over two decades [3, 4], limitations [24] of the indicators have seen modifications of the assessment methodologies on many levels [25]. Fourteen countries have similar assessment systems to Australia and New Zealand, but none of them is a developing country [14]. There have been no studies in low-resource settings as to how research performance should be measured, although bibliometric indicators and Google Scholar were argued as appropriate [26, 27]. Cole et al. [28] identified a limited number of indicators of research outputs and outcomes from a review of 12 capacity-building evaluations in LMICs. It is ironic that there should be a multitude of tools and indicators to measure the quality of health services in LMICs, but there are no metrics to measure research productivity [29].

Our study, therefore, utilising a Delphi survey, which is a validated method of obtaining consensus [21], has identified a composite of research processes and outcomes that could be used to assess research performance in LMIC settings. Indicators such as research collaborations, national recognition, supervision of researchers, and contribution to the research environment were ranked high and were not components of bibliometric indices. The number of publications was ranked 8th and citations 11th in importance for the LMIC setting, whereas the same were ranked 3rd and 7th, respectively, in their importance in the high-resource setting. Other process markers of research activity were identified, such as the implementation of research findings, impacting change in practice and interacting with the media. It is possible that the favourable ranking of process and practice-orientated research indicators over bibliometric ones could be due to the majority of clinical and epidemiological experts on the Delphi panel.

It is quite possible that research clinicians in LMIC settings can be assessed as high performing clinical researchers, without publishing a paper, by adapting evidence published elsewhere to develop locally applicable clinical guidelines, making sure the guidelines are followed by performing a clinical audit, and then presenting the findings at a conference and disseminating the findings nationally. The outlined scenario and research performance measures may appeal to busy clinicians who are already in practice in LMIC settings as they have limited time, do not have research skills, and/or have no access to research support.

The undercurrent theme from the expert panel was for clinical researchers in Pacific Island countries to collaborate in research, lead research, promote research and translate evidence to practice. It was obvious from the survey that there was a dichotomy in emphasis for researchers in high-resource countries and clinical researchers in LMIC settings. Research program funders and evaluators in LMICs agree that outcomes should be the development of research skills, development of sustainable collaborations, time spent on research, funding obtained, invitations to speak, research in conference proceedings, and membership in professional societies [28]. There was no ranking or value given to each item on the list and there was no mention of publications in peer-reviewed journals.

Engaging local clinical researchers, as we have done, in the process of determining research indicators engenders buy-in and ensures local context and values are acknowledged in the assessment methodology [23]. Research performance measures for Pacific clinicians should address both processes and outcomes. Although they were uncertain initially of what research indicators were, they were quick to make an association with the educational and professional tasks that they collect for professional development points. The clinicians were very aware that their main role is of clinicians and, due to the limited time for research, they wanted to maximise the number of research points they could obtain from performing tasks that were part of their clinical work. Therefore, a balance is needed, as articulated by a panellist: “*Research is a task that has universal principles that experts who are not clinicians can speak to in ways that clinicians may not. Clinicians have hands-on insight into the clinical context of their research and so offer the exercise of developing and assessing performance indicators from a more ‘grounded’ perspective. Together these two perspectives ought to provide a richer, more nuanced set of indicators for Pacific health researchers in resource poor countries*”.

The Pacific Island clinicians in the BRRACAP Study had moderate success compared to another research capacity-building course of 24 Pacific Island clinician participants [30]. Bissell et al. [30] reported on two research courses, each with three 5-day modules; assessment of 24 participants at the end of 2 years found that 17 (71 %) completed the course and 18 manuscripts had been accepted for publication in peer-reviewed journals. In our study [20], comprised of a workshop of 7 days and supportive mentoring, only 18 (64 %) returned the assessment questionnaire and there were only two accepted publications. The participants’ clinical background was similar in both studies, although the majority of Bissell’s participants were from Fiji; both groups had access to experienced research mentors and online support. The better success of Bissell et al. [30] is most likely due to a course structure that was modular in nature, lasting a total of 3 weeks and with assignments in between. Their focus was also on publications, whereas that of the BRRACAP Study was on clinical research/audit and teamwork. Another research capacity-building program saw 23 Fellows from Asia publish five (22 %) Cochrane reviews after a median of 4 weeks attachment in Australia [31]. Whereas Bissell et al. [30] made a good argument for publications as an endpoint, no other study has had the same success in the number of publications. A survey in Pakistan of 54 overseas trained doctorate recipients 15 years post-training found that only 66 % had published internationally [13].

Not all research-building programmes or workshops have similar aims and, therefore, similar results to those focused on publications. For example, the course by Bates et al. [32] consisted of two 1-week workshops and had effectiveness measures that included process, content and outcome indicators (which did not include publications), which included a Diploma. Other research workshops, with a similar duration to ours but with different objectives and outcome measures [32-36], looked at different outcomes of which publications were not a primary outcome. The panellists identified extra research indicators as national recognition and being first authors of publications. National recognition can be achieved by research advocacy and championing the development and dissemination of practice guidelines. The emphasis in indigenous researchers being first authors of papers may be due to the advocacy over the years [15, 37]. The panellists ranked publications eighth in importance preferring translation of research findings, dissemination and collaborations as more important for clinicians in an LMIC setting. In that regard, 7 (39 %) of the BRRACAP Study participants performed 10 or more tasks from a list of 21 research indicators.

A limitation of our study was that the BRRACAP Study participants’ replies to the survey were not verified with the exceptions of the publications. In addition, the views of 28 reproductive health clinicians from six LMICs may not be generalizable to all LMICs. A weakness in our study was that we did not conduct a similar Delphi survey for managers, professional organisations and funders of research in the Pacific countries. This would have been important, as the participants had identified their respective professional organization as the entity that should perform their research assessment, and may have ranked the research indicators differently, as LMICs tend to invest in human resources and infrastructure preferring funders to fund projects [38].

## Conclusions

Research performance assessments need to acknowledge the resource context of those being measured and engage the participants in a dialogue as to which indicators or assessment tools should be used. Ranking by a panel of experts has identified the most important indicators for clinicians working in the Pacific Islands – a low-resource setting – and these include translating research evidence into practice, dissemination and collaboration. Clinicians should not be assessed on publication output alone unless they have had the necessary training and barriers, such as time commitment, appropriately addressed and a research environment that is supportive of nurturing research and researchers.

## Additional files

Additional file 1:
**Delphi Questionnaire 1: Determining research performance activity or indicators for pacific clinical researchers [** 38 **].** (DOC 73 kb) Additional file 2:
**Delphi Questionnaire 2: Determining research performance activity or indicators for pacific clinical researchers.** (DOC 156 kb) Additional file 3:
**Questionnaire for BRRACAP Study participants: Review of research/audit performance activity.** (DOC 30 kb)
