# Supplementary material for: Development and use of a research productivity assessment tool for clinicians in low-resource settings in the Pacific Islands: a Delphi study
Source: Health Res Policy Syst. 2016 Jan 29;14:9. doi: 10.1186/s12961-016-0077-4 (PMC4732024; doi:10.1186/s12961-016-0077-4)
Supplement: Additional file 1: — Delphi Questionnaire 1: Determining research performance activity or indicators for pacific clinical researchers [ 38 ]. (DOC 73 kb) [file 12961_2016_77_MOESM1_ESM.doc]

**Additional file 1:** Delphi Questionnaire 1: Determining Research Performance Activity or Indicators for Pacific Clinical Researchers

Thank you for agreeing to take part in this “panel of experts consultation” using the modified Delphi process in two rounds of questionnaires. The aim is to produce good research performance/acivity indicators for clinicians in the Pacific Islands, based on “CREAM” which stands for clear, relevant, economic, adequate and monitorable.

The questionnaire is in 5 parts from A-E and includes the perfomance indicators as were determined by the clinicians themselves. We would appreciate if you could comment on them.

**QUESTIONS:**

**A. Evaluation tool construction**

How important is it to you that:

| Evaluation tool construction | Very Important | Somewhat Important | Somewhat Unimportant | Not at all Important |
| --- | --- | --- | --- | --- |
| The performance indicators identified by the clinicians themselves be used in their evaluation? |  |  |  |  |
| The performance indicators should be a hybrid of both expert and Pacific clinician perspectives |  |  |  |  |
| The performance indicators as identified by the expert panel should be the only one used |  |  |  |  |

**B. Academic relevance**

As an academic, how relevant are these to you?

| Specific research performance indicators are used to assess performance of academics in developed countries. (They are not arranged in any order). | Very relevant | Somewhat Relevant | Somewhat Irrelevant | Not at all Relevant |
| --- | --- | --- | --- | --- |
| Number of publications |  |  |  |  |
| Conference presentations |  |  |  |  |
| Article Citations |  |  |  |  |
| Books published |  |  |  |  |
| Research collaborations |  |  |  |  |
| Research supervision |  |  |  |  |
| Creative works |  |  |  |  |
| Reviewer |  |  |  |  |
| Peer Esteem e.g. journal editor |  |  |  |  |
| Contribution to the research environment e.g. research meetings |  |  |  |  |
| Research funding received |  |  |  |  |
| International recognition |  |  |  |  |
| National recognition |  |  |  |  |

**C. Pacific relevance**

You have had research experience in the Pacific.

How relevant are these for Pacific clinicians or those who do part time research in a low resource setting?

| Specific research performance indicators are used to assess performance of academics in developed countries. (They are not arranged in any order). | Very relevant | Somewhat Relevant | Somewhat Irrelevant | Not at all Relevant |
| --- | --- | --- | --- | --- |
| Number of publications |  |  |  |  |
| Conference presentations |  |  |  |  |
| Article Citations |  |  |  |  |
| Books published |  |  |  |  |
| Research collaborations |  |  |  |  |
| Research supervision |  |  |  |  |
| Creative works |  |  |  |  |
| Reviewer |  |  |  |  |
| Peer Esteem e.g. journal editor |  |  |  |  |
| Contribution to the research environment e.g. research meetings |  |  |  |  |
| Research funding received |  |  |  |  |
| International recognition |  |  |  |  |
| National recognition |  |  |  |  |

**D. Other Indicators**

What other indicators or markers of research activity do you think we need to use for clinicians in a low resource setting such as those in the Pacific?

Please list and clarify if needed:

| **Indicators** | **Clarification** |
| --- | --- |
|  |  |

**E: Research performance indicators by the Pacific clinicians**

The following were the indicators identified by the clinicians themselves during a focus group sessions. Please comment on their relevance.

**Midwives/Nurses**

| **Performance Indicators** | **Comments** |
| --- | --- |
| Organizing research meetings |  |
| Teaching or mentoring research students |  |
| Interacting with mentor |  |
| Successful at obtaining research funding |  |
| Submit a research proposal |  |
| Presentation at a regional research conference |  |
| Completion of a clinical audit project |  |
| Regional recognition as a researcher |  |
| Research publication in a peer reviewed journal |  |
| Lead author of a research based practice guideline that is endorsed or approved |  |
| Participation in journal clubs |  |

**Medical/ Doctors**

| **Performance Indicators** | **Comments** |
| --- | --- |
| Presentation at a regional research conference |  |
| Participation in journal clubs/CME meetings/ Perinatal meetings |  |
| Organizing research meetings |  |
| Attending research conference |  |
| Successful at obtaining research funding |  |
| Annual Report Writing and Recommendation |  |
| Write new guidelines is endorsed or approved |  |
| Research publication in a peer reviewed journal |  |
| Submitting a research proposal |  |
| Completion of a clinical audit project |  |
| Revising guidelines/protocols |  |
| Teaching or mentoring research students |  |
| Interacting with mentor |  |

Other comments:

Please email your comments/feedback to Dr Ekeroma by the 30 August 2014.

Your name: _________________________________________

Thank you for completing the questionnaire. Your responses to this survey will be kept **confidential**.

APPROVED BY THE UNIVERSITY OF AUCKLAND HUMAN PARTICIPANTS ETHICS COMMITTEE ON 25 September 2012..FOR (3) YEARS REFERENCE NUMBER 8373.
