# Supplementary material for: Development and use of a research productivity assessment tool for clinicians in low-resource settings in the Pacific Islands: a Delphi study
Source: Health Res Policy Syst. 2016 Jan 29;14:9. doi: 10.1186/s12961-016-0077-4 (PMC4732024; doi:10.1186/s12961-016-0077-4)
Supplement: Additional file 2: — Delphi Questionnaire 2: Determining research performance activity or indicators for pacific clinical researchers. (DOC 156 kb) [file 12961_2016_77_MOESM2_ESM.doc]

**Additional file 2:** Delphi Questionnaire 2: Determining Research Performance Activity or Indicators for Pacific Clinical Researchers

Thank you for completing the Delphi Questionnaire 1 in this “panel of experts consultation.” There are 19 of you - which is a good sample for a Delphi survey. You have all either performed research or led research teams in the Pacific Islands. Eight of you have professorial appointments. Ten are based in Australia, seven in New Zealand, one from Papua New Guinea and one from the United States.

In this second questionnaire, we have summarised the findings of the first questionnaire under emerging themes and ranked the research performance indicators as they were indicated in the first questionnaire. Discussions generated for each identified indicator are given. We have combined the indicators for midwives and doctors as there were no major differences identified between the two lists.

Your name: _________________________________________ Your responses will be kept confidential.

**QUESTIONS:**

**A. Academic relevance and Pacific relevance**

Research indicators, as used by academic institutions in developed countries were listed and the ‘panel of experts’ were asked to rank on a 4 point scale (from very relevant to not relevant), their relevance to themselves personally and then their perspective on their to those clinicians who work in low resource settings.

Ranked by the ‘panel of experts’ in their order of importance. All indicators that had a ranking score of <2.50 are not listed.

| Known research performance indicators for academics in developed countries | Academic relevance  Ranking score | Known research performance indicators for academics in developed countries | Pacific relevance Ranking score |
| --- | --- | --- | --- |
| 1. Research collaboration | 3.88 | 1. Research collaborations | 3.76 |
| 2. Research supervision | 3.65 | 2. National recognition | 3.65 |
| 3. National recognition | 3.24 | 3. Research supervision | 3.35 |
| 4. Number of publications | 3.24 | 4. Contribution to the research environment | 3.35 |
| 5. Contribution to the research environment | 3.18 | 5. Conference presentations | 2.82 |
| 6. International recognition | 3.12 | 6. Research funding received | 2.59 |
| 7. Article citation | 3.06 | 7. International recognition | 2.53 |
| 8. Reviewer | 2.88 |  |  |
| 9. Conference presentations | 2.82 |  |  |
| 10. Research funding received | 2.82 |  |  |
| 11. Peer esteem | 2.71 |  |  |

There are only seven research indicators in Pacific relevance as none of the other indicators were ranked highly (e.g. number of publications).

**Final comments or re-ranking of indicators for Pacific relevance:**

**B: Research performance indicators from the Pacific clinicians**

A group of Pacific clinicians (medical and midwifery/nursing) identified the following research indicators during focus group sessions in 2013. The indicators for nurses and medical doctors have been combined as there was significant cross-over and similarities. Their ranking of importance was based on the number of research points they should receive for every indicator/activity.

The list appear in order of importance deduced from the comments of the ‘expert panel’. Comments below the ranking were by the expert panellists and only comments that were more than just two words are listed.

I have arbitrarily ranked these from 1 to 10, with 10 being the factor that had the most approval from the ‘expert panel’. If you were to give points out of 10 for each completed activity, what point will you award?

**1. Presentation at a regional research conference**

| Rank by the expert panel | Rank by the Pacific clinicians | New ranking in points |
| --- | --- | --- |
| 10/10 | 7.5 points |  |

- Important to share what is happening in the region.
- Crucial – gives ownership and pride
- Important for networking or disseminating results locally
- Very relevant as local knowledge facilitates conveying the findings in context and encourages other Pacific ‘would be ‘ researchers and others to consider research rather than seeing research as arcane
- Clinicians should always try to be present and make contributions to regional research conferences
- Yes, useful knowledge transfer and idea sharing.
- Critical- relationships are key to success in low resource environments and networking at conferences can facilitate these relationships. However, attendance at these events need to be targeted to the most relevant person, not just the person with the highest status or the person who is thought to deserve a reward of an international trip.

**2. Research publication in a peer-reviewed journal**

| Rank by the expert panel | Rank by the Pacific clinicians | New ranking (if appropriate) |
| --- | --- | --- |
| 9/10 | 10 points |  |

- Important, provides the evidence for others in the Region
- Fully agree – this is one from of peer-review that is important, alternatively, it is possible to invite quality peer reviews through other means.
- The publications to be made available and understood by local workers. Regional and local journals could potentially be ‘snobbed’ for high impact factor journals which a number of Pacific workers cannot access.
- This is the main indicator – especially as first author.
- This is important and will require collaboration and support.
- Yes, the key means of honing research skills as well as dissemination to effect change. Publication is not easy for most peer reviewed journals so indicates a certain standard.
- Important, provides the evidence for others in the Region.
- Very important- this brings opportunities for national and international collaborations and is often the way an applicant is assessed for grants.
- This is important and will require collaboration and support.

**3. Successful at obtaining research funding**

| Rank by the expert panel | Rank by the Pacific clinicians | New ranking (if appropriate) |
| --- | --- | --- |
| 8/10 | 10 points |  |

- Not so important – can be a “detractor” if unsuccessful.
- Needed to support infrastructure to do research.
- Agree but will often require support from national and/or international colleagues.
- Very valuable but often a challenge early on as a lead applicant.
- This will need to be in collaboration with others – in all countries – not just lower resource countries.
- Yes, a key indicator of expertise.
- Very important - this allows control of the research agenda to stay with those leading in the low-resource setting.
- Important skill and contribution.
- For local or national funds this seems attainable. For international funds this may be unlikely.

**4. Submitting a research proposal**

| Rank by the expert panel | Rank by the Pacific clinicians | New ranking (if appropriate) |
| --- | --- | --- |
| 7/10 | 10 points |  |

- Can be tricky especially without back up.
- Of some value
- Needed for funding
- Fully agree however, participating with others in a team effort should be included here.
- Priorities on ‘operational research’? Although international standards should not be compromised.
- Very valuable but a bit further down the track and not so many opportunities in the Pacific.
- Yes, but it may be a bad proposal – winning the funding is a better indicator of research quality.
- Important skill and contribution; is underestimated and should be recognized in terms of time input and identifying more rigorous mechanisms for investigating issues.
- For solo work may be hard. But in collaboration with research scientists as collaborators (both local and international) somewhat relevant.

**5. Writing or revising clinical guidelines**

| Rank by the expert panel | Rank by the Pacific clinicians | New ranking (if appropriate) |
| --- | --- | --- |
| 6/10 | 10 points |  |

- This must always be in collaboration with the nurses and midwives. It is not only the doctors who write guidelines.
- Very relevant especially local guidelines.
- Important for implementation of best practice.
- Fully agree (based on research reported from elsewhere, from the region or from own work.
- Very relevant. Political endorsement.
- I suspect these are mainly translated rather than built from scratch.
- So long as based on systematic lit review and try to get summary of guideline published in journal.
- Good to ensure current context.
- Skills needed in these areas to ensure high quality.
- Very relevant and especially guidelines for primary care level and nursing care.
- By invitation – best to have national or at least regional guidelines – every clinician should not be producing his own.
- This is important and will require collaboration and support.

**6. Completing a clinical audit project**

| Rank by the expert panel | Rank by the Pacific clinicians | New ranking (if appropriate) |
| --- | --- | --- |
| 5/10 | 5 points |  |

- Important for best practice
- Valuable and should be recognized
- Yes, partially, although research and audit are not the same.

**7. Teaching or mentoring research students**

| Rank by the expert panel | Rank by the Pacific clinicians | New ranking (if appropriate) |
| --- | --- | --- |
| 4/10 | 1 point per hour |  |

- Training the next generation is vital.
- Very relevant for research in the clinical setting
- Important but often not possible due to lack of support and capacity.
- Yes, a fair indicator although one may do this badly – productivity of students (e.g. publication) would be a better index, although still not guaranteed to reflect supervisor research excellence.
- Very important from a sustainability perspective.
- Important contribution – if mentoring research activity as opposed to general mentoring, then this is relevant.
- Important but often not possible due to lack of support and capacity.

**8. Organising research meetings**

| Rank by the expert panel | Rank by the Pacific clinicians | New ranking (if appropriate) |
| --- | --- | --- |
| 3/10 | 10 points |  |

- Meetings take individuals away from their regular work – resource capacity is always an issue in the Pacific. In-country research meetings could be appropriate.
- Important for building research capacity.
- Relevant although internet based meetings should be seriously considered given the geographic and logistic problems of travels in the Pacific (cost and time).
- This is important to participate in – usually a committee function.
- The best local clinician in this area should organize this activity.
- Somewhat worthwhile so long as not merely an administrative role; if thinking carefully about structure and format to grow research then useful.
- Very important - I would suggest organizing and leading research meetings to ensure that the research agenda remains under the leadership of people from that place (and not the agenda of international researchers alone).
- This is important. Many will need support to do this.

**9. Participation in journal clubs/CME meetings/ Perinatal mortality meetings**

| Rank by the expert panel | Rank by the Pacific clinicians | New ranking (if appropriate) |
| --- | --- | --- |
| 2/10 | 1 point per hour |  |

- Crucial – sets the tone of a unit.
- Useful contribution; more important if organizing it.
- Excellent for gaining research skill/mentoring.
- This is an indicator of attendance, not necessarily of research/audit capacity.
- Good to stimulate research ideas and critical thinking about data quality, trends etc.
- Very important- maximum benefits if suitable mentors/research leaders can also participate.
- Valuable as a self-educational process, unless playing a role in organizing and structuring the club.
- Very good to do and not too hard.

**10. Attending research conference**

| Rank by the expert panel | Rank by the Pacific clinicians | New ranking (if appropriate) |
| --- | --- | --- |
| 1/10 | 1 point per hour |  |

- Important to understand what is happening in the region.
- Relatively insignificant.
- For networking and collaboration, disseminating knowledge.
- Absolutely! Especially the follow up of Recommendations and its evaluation for relevance with time and progress of projects and national and regional capacity and relevance.
- Less valuable – researchers need to present.
- Mere attendance is not enough, presenting at research conference or attending workshops.
- Easy to attend and be passive - only really useful if presenting or engaged in research meetings as an investigator.

**C: Other Indicators for low resource countries**

The ‘expert panel’ have indicated other possible research indicators (different from those mentioned above) that could be used to review the research performance of clinicians in low resource settings. Some of these can be interpreted as performance indicators for training institutions and health departments.

Please indicate with a tick below each proposed indicator what you deem as their relevance.

| **Indicators** | | **Clarification** |
| --- | --- | --- |
| 1. Impact or change as a result of research | | Resulting from research  Immediate relevance to practice or environment  Improved systems through use of research results  How does the research contribute to improvements in clinical practice/public health practice.  Effecting improvement in practices and policies is a priority. |
| Very relevant |  |
| Somewhat relevant |  |
| Somewhat not relevant |  |
| Not relevant |  |
| 2. Implementation/Translation of research findings | | Research to inform improved health outcomes in local settings (I think this is important in all settings, but especially so where there is a high burden of both infectious diseases and NCDs).  Leadership in pushing research evidence into the policy arena. This is a real sticking point for all researchers – we are good at collecting data, but leave it there and it is not good sitting in journals in contexts where that evidence may play a role in promoting safer, more effective, equitable etc., practice or environments.  There is a lot of research already done – the challenge is getting it into practice. This could be in policy documents, practice protocols etc. Evaluations of this translational research is important.  Local evidence generated relevant to local settings is especially important for informing locally-relevant health policy. This reduces the risk of imported responses that ‘miss the mark’ in terms of local cultural, social and spiritual understandings of health. |
| Very relevant |  |
| Somewhat relevant |  |
| Somewhat not relevant |  |
| Not relevant |  |
| 3. Inter-disciplinary research collaborations  Number of active regional collaborations | | In context where resources are limited, the collaboration between disciplines (e.g. education and health) can make an important contribution to the overall health of the community.  Participation with national and regional colleagues in formulating important research questions, conducting studies and transforming practice, programs and policies.  Indicator of activity involving a wider group with mix of expertise required to address major LMIC MDG-relevant issues. |
| Very relevant |  |
| Somewhat relevant |  |
| Somewhat not relevant |  |
| Not relevant |  |
| | 4.Community engagement and recognition | |  | | --- | --- | --- | | Very relevant |  |  | | Somewhat relevant |  | | Somewhat not relevant |  | | Not relevant |  | | | Experience engaging community/ies that research targets.  Recognition received from community/ies that research targets. |
| 5. Position as PI/Leadership in research design and priorities | | Pacific clinicians taking the initiative and leading new research initiatives. This will mean investment in research methods or translation and interpretation of research to benefit the field and the clinicians career development.  Pacific clinicians involvement in research planning, priority setting and research design – both at a project level and at a research governance level (e.g. involvement in ethics committee, research councils etc).  Need to identify contributions to study design, measures, processes and role in co-authored publications. As opposed to merely facilitating or ‘opening doors’ for outsiders. |
| Very relevant |  |
| Somewhat relevant |  |
| Somewhat not relevant |  |
| Not relevant |  |
| 6. Lead authorship on peer-reviewed papers and other dissemination | | Pacific clinicians to take greater role in the preparation of journal or conference and other forms of presentation – this may require additional support and training as writing and presentation not traditionally part of clinical role. |
| Very relevant |  |
| Somewhat relevant |  |
| Somewhat not relevant |  |
| Not relevant |  |
| 7. Advocacy | | Clinicians are still highly respected in society (Pacific and non-Pacific) and their voice is not heard enough. Free media, working with other researchers (being realistic about time and capacity constraints) and developing a track record in a field is vital as part of the wider efforts for change and development in the clinical and public health fields. |
| Very relevant |  |
| Somewhat relevant |  |
| Somewhat not relevant |  |
| Not relevant |  |
| 8. Visiting scholars to the department or to the researcher | | Visiting scholars can grow the enthusiasm for research grows |
| Very relevant |  |
| Somewhat relevant |  |
| Somewhat not relevant |  |
| Not relevant |  |
| 9. Engagement with policy makers, health authorities, and other relevant administrative structures | | Do clinicians interact with policy and decision-making structures; do they take research forward and aim to influence practice? |
| Very relevant |  |
| Somewhat relevant |  |
| Somewhat not relevant |  |
| Not relevant |  |
| 10. Publication of policy briefs or other accessible research summaries for the public and for policy makers. | | Beyond formal publication… do researchers produce other types of outputs that are more accessible to a broader community? |
| Very relevant |  |
| Somewhat relevant |  |
| Somewhat not relevant |  |
| Not relevant |  |
| 11. Media interaction – writing for mainstream media – such as op-eds – plus interviews and other public presentations and interviews. (Altmetric metrics). | | Other forms of engagement and publication, which are directed at a more popular and general audience.  Measure of social media and other interactions with published materials. |
| Very relevant |  |
| Somewhat relevant |  |
| Somewhat not relevant |  |
| Not relevant |  |
| 12. Relevance of research | | The relevance and importance of the research questions to the setting in which the person works and in the Pacific (this indicator is related to the next two indicators) -  The extent to which the research contributed to clinical practice, programs and policies the setting in which the person works and in the Pacific  The extent to which the researcher used the research results to convince decision-makers to change or develop innovative practice, programs and policies |
| Very relevant |  |
| Somewhat relevant |  |
| Somewhat not relevant |  |
| Not relevant |  |
| 13. Research Leadership | | Recruiting promising clinicians and researchers from the region as well as from other countries to collaborate in research activities and for transforming the results into better practice and policies. |
| Very relevant |  |
| Somewhat relevant |  |
| Somewhat not relevant |  |
| Not relevant |  |
| 14. Development of Pacific or ethnic specific standards validated using Pacific ethnic measures | | Specific biomedical or physiological standards specific to Pacific ethnic groups validated compared to the ‘usual’ global standards eg. Lung functions, childhood development milestones, IQ, BMI- SF |
| Very relevant |  |
| Somewhat relevant |  |
| Somewhat not relevant |  |
| Not relevant |  |
| 15. Proportion of 1) paid effort, and 2) overall time spent in all research activities | | I suspect the everyday environment of Pacific health researchers does not allow individuals to spend much time or overall effort in leading research and contributing innovative ideas, insights and techniques. |
| Very relevant |  |
| Somewhat relevant |  |
| Somewhat not relevant |  |
| Not relevant |  |
| 16. Amount of funds made available by the national or regional Pacific institutions to researchers | | If we expect Pacific researchers to conduct research we must understand how available national and regional funds are to them for their own pilot work. |
| Very relevant |  |
| Somewhat relevant |  |
| Somewhat not relevant |  |
| Not relevant |  |
| 17. Workforce capacity | | Availability of students and trainees to assist in research |
| Very relevant |  |
| Somewhat relevant |  |
| Somewhat not relevant |  |
| Not relevant |  |
| 18. Quality assurance projects or Clinical Audit | | These are often the precursor to doing research and so should be supported and encouraged.  Especially perinatal and maternal morbidity (labour ward performance – partograph audit for example) and maternal mortality. |
| Very relevant |  |
| Somewhat relevant |  |
| Somewhat not relevant |  |
| Not relevant |  |
| 19. Hand over (morning ) meetings to review the (previous day/night) activity and to invite critique of management of ‘difficult’ patients. | | Most of our clinicians do not engage sufficiently in case discussion and management critique |
| Very relevant |  |
| Somewhat relevant |  |
| Somewhat not relevant |  |
| Not relevant |  |
| 20. Evaluations of innovative practices | | Again, these are often the precursor to doing research and so should be supported and encouraged. |
| Very relevant |  |
| Somewhat relevant |  |
| Somewhat not relevant |  |
| Not relevant |  |
| 21. Productivity of group, not just an individual researcher | | Group ideal for sustained research productivity and better aligned with Pacific values of collective effort. |
| Very relevant |  |
| Somewhat relevant |  |
| Somewhat not relevant |  |
| Not relevant |  |

**D and E sections are for your information only. They are responses/summaries from the first questionnaire.**

**D. Evaluation tool construction.**

All the 17 members of the expert panel agreed that the research indicators for clinicians working in the Pacific or low resource settings should be a hybrid of both those identified by the clinicians and those identified by the expert panel.

Some of the reasons given were:

They should be based on both evidence from research of what is valid as an indicator, *and* on relevance to the local context (with input from clinicians ). Given the challenges many LMIC clinicians face with isolation, communications, access to library/internet resources etc, this needs to be taken into account.

A combination as they need to be responsive to the context and the capacity of the country and the individuals.

If one asked most ordinary clinicians they would just fumble around I think.

Neither has the full perspective.

A hybrid is needed to take into account the daily life of being a clinician in the Pacific and the general evaluation metrics that working researchers use.

A hybrid of both – clinicians may not be aware of all potentially useful performance indicators. It is good to share indicators across disciplines.

No one group has all the answers. Many possibilities are created when groups openly share ideas and experiences.

Research is a task that has universal principles that experts who are not clinicians can speak to in ways that clinicians may not. Clinicians have hands-on insight into the clinical context of their research and so offer the exercise of developing and assessing performance indicators from a more ‘grounded’ perspective. Together these two perspectives ought to provide a richer, more nuanced set of indicators for Pacific health researchers in resource poor countries.

Need to address whole of system, as they interrelate need to do both.

A hybrid of both as although clinicians bring a practical and logistic, and ideally patient-centred focus to performance indicators, a domain / disciplines needs to invite input from allied professional who bring an alternative or complimentary perspective that can be useful for driving change.

You need experts to guide the clinicians with the structure and the content.

It needs to reflect both objective and subjective insights. However, it does depend on what use is being made of these indicators – is it for peer assessment or is it for some external forms of recognition and e.g. promotion? If the former, peer assessed would be fine; if the latter – then hybrid would make more sense.

Clinicians can identify indicators important for the local settings as markers of performance that external experts may under-estimate. This way, they have ownership of the indicators and a stronger commitment to achieve them.

Experts should be invited to give their opinions on what the clinicians select first, and then offer OTHER suggestions for consideration by the local clinician group.

A hybrid of both, clearly need something that Pacific clinicians have identified as relevant and needs to match with what is recognised internationally.

I think the 2 sets have a number of overlaps so most key indicators are covered. I think the need to translate research into policy and practice should drive some of the indicators that are identified by clinicians, such as writing guidelines etc. In some ways the short term research needs in the pacific are more operational so the types of outputs could well be guidelines/policies that are published in local journals (non-peer reviewed).

**E. Other comments:**

There is a growing body of literature on Operational Research (OR) in LMICs and the work of The Union and MSF in this area. OR seeks to develop relevant research to health services in low resource settings using routinely collected data. I have taught on courses in the Pacific using this model. The key metric is publication and justification for this is argued in the OR literature.

Should include whole of workforce as they will be better together rather than separated.

Whole of system reform is required.

I like the thinking behind the project.

I expect the major limitations for conducting research is the heavy clinical load that allows relatively little time for what some clinicians consider ‘research’ to be a relative luxury. Furthermore, the task of attracting adequate financial resources and technical assistance to conduct research in the region must be extremely challenging. Any initiative to train clinicians in research must be linked with ensuring they are given active support and encouragement through these resources to plan and conduct research. Training in research without increasing ‘supply’ is unlikely to increase’ demand’ by clinicians to contemplate meaningful and effective research activities.

Hope this can identify the right hybrid of research/audit indicators.

I think the wrong indicators leads to a shift in focus away from the priority areas. The priority areas from my experience stem from a need to understand the local context of disease occurrence in terms of the causal determinants and institutional/technical responses. There are a large number of gains to be made through simple operational research that will lay the foundation for more in depth and sophisticated research programs once local staff become more skilled. It is essential that the indicators are set to encourage progression of research-interested staff through the various levels needed to attain a high level of skill and expertise in research.

**Thank You**
