# Supplementary material for: Development and use of a research productivity assessment tool for clinicians in low-resource settings in the Pacific Islands: a Delphi study
Source: Health Res Policy Syst. 2016 Jan 29;14:9. doi: 10.1186/s12961-016-0077-4 (PMC4732024; doi:10.1186/s12961-016-0077-4)
Supplement: Additional file 3: — Questionnaire for BRRACAP Study participants: Review of research/audit performance activity. (DOC 30 kb) [file 12961_2016_77_MOESM3_ESM.doc]

**Appendix 3:** Questionnaire for BRRACAP Study participants: Review of Research/audit performance activity

**The BRRACAP Study – Building Reproductive Research and Audit Capacity and Activity in the**

**Pacific Islands**

Please outline the research/audit activities that you have made **since the research workshop in March 2013.** The questionnaire has 22 questions and should take about 20 minutes to complete. Please circle appropriate Yes/No/Not Applicable and give details if it’s a Yes answer.

1. Tell us about any local or regional research meeting you organized? Yes No NA
   If Yes, please give details:
2. Were there opportunities to teach research to medical, nursing or midwifery students? Yes No NA

If Yes, please give details:

1. Were there conversations/discussions with your research mentor?

Yes No NA

If Yes, please give details how you had the conversation and how many times

If Yes, was the mentor helpful? Yes No

1. Was there an opportunity to supervise a research project? Yes No NA

If Yes, please give details:

1. Did you start an audit project? Yes No NA

If Yes, please give details:

If Yes, was the audit project completed Yes No

1. Did you submit a research proposal for funding? Yes No NA
   If Yes, please give details:
2. What funding did you receive for your research? Yes No NA

If Yes, please elaborate:

1. Was there an opportunity to present a research paper at a local or regional research meeting? Yes No NA

If Yes, please give details:

1. Was there an opportunity to attend a local or regional research conference/meeting? Yes No NA
   If Yes, please give details:
2. Were you part of a research collaboration? Yes No NA

If Yes, please elaborate:

1. Did you submit a research proposal to an Ethics Committee? Yes No NA

If Yes, please detail:

1. Were you recognized by your peers as a clinical researcher? Yes No NA

If Yes, please elaborate:

1. Did you receive an award/certificate/qualification in research? Yes No NA

If Yes, please detail:

1. Did you publish a paper in a peer-reviewed journal? Yes No NA

If Yes, please detail:

1. Did you review a paper for publication? Yes No NA

If Yes, please elaborate:

1. Was there an opportunity to revise a clinical guideline? Yes No NA

If Yes, please detail:

1. Did you assist in writing a new guideline? Yes No NA

If Yes, please give details:

1. Did you take part in a journal club? Yes No NA

If Yes, please detail:

1. Did you change the way you practice as a result of research evidence? Yes No NA

If Yes, please detail of give an example:

1. Did you advocate for changes in clinical practice or a policy as a result of research evidence? Yes No NA

If Yes, please detail:

1. Did you have an interaction with your managers or with the media as a result of research evidence? Yes No NA

If Yes, please detail:

1. Did you engage with the local community about a research project? Yes No NA

If Yes, please elaborate:

Thank you for taking part. Those who complete and return the questionnaire will be informed of the overall results of the questionnaire by email.

Name _______________________________________________________________

Signature ________________________________________________ Date:_________________

APPROVED BY THE UNIVERSITY OF AUCKLAND HUMAN PARTICIPANTS ETHICS COMMITTEE ON 25 September 2012..FOR (3) YEARS REFERENCE NUMBER 8373.
